# Advantages of intraoperative implantation of Impella 5.5 SmartAssist in the Management of Acute Post-Infarction Ventricular Septal Defect with cardiogenic shock

**DOI:** 10.1186/s13019-021-01513-y

**Published:** 2021-05-17

**Authors:** Jeko M. Madjarov, Michael G. Katz, Svetozar Madzharov, Shahood Fazal, Francis Robicsek

**Affiliations:** 1 Sanger Heart and Vascular Institute/ Atrium Health, Charlotte, NC USA; 2 Department of Cardiology, Cardiovascular Research Institute, Icahn School of Medicine at Mount Sinai, 1470 Madison, Ave, Box 1030, New York, NY 10029-6574 USA

**Keywords:** Ventricular septal defect, Impella 5.5 SmartAssist, Acute myocardial infarction, Cardiogenic shock

## Abstract

**Background:**

Despite advances in surgical techniques and aggressive therapy of post-infarction ventricular septal defect (VSD) with cardiogenic shock, the overall morbidity and mortality is frustratingly high. The Impella 5.5 SmartAssist (Abiomed, Danvers, MA) is a surgically implanted temporary device, recently approved by the FDA (https://www.businesswire.com/news/home/20190925005454/en/) for treatment of patients in cardiogenic shock, and may fill a technological gap for patients who require acute circulatory support after VSD closure.

**Case presentation:**

We report our initial experience for two patients with post myocardial infarction VSD in the setting of cardiogenic shock supported with trans-aortic implantation Impella 5.5 SmartAssist. First patient had a posterior VSD with a left to right shunt (Qp/Qs ratio of 3.3), blood pressure 80/35 mmHg, right ventricle dysfunction, severe pulmonary arterial hypertension (an estimated systolic pulmonary artery pressure of 45 mmHg), and severe mitral valve regurgitation. Second patient was admitted for massive MI with large anterior VSD (Qp/Qs ratio of 2.8). Under cardiopulmonary bypass with cardioplegic arrest both patients underwent urgent VSD closure with trans-aortic implantation of the Impella. Minimal postoperative support was required. Patients were discharged on postoperative day 10 and 14 and remained well 3 months later. Follow-up echocardiogram showed no residual shunt.

**Conclusions:**

Early surgical implantation of Impella 5.5 SmartAssist can prevent multiorgan dysfunction and stabilize the patients in cardiogenic shock with post-myocardial infarction VSD.

**Supplementary Information:**

The online version contains supplementary material available at 10.1186/s13019-021-01513-y.

## Background

**Figure 1:**
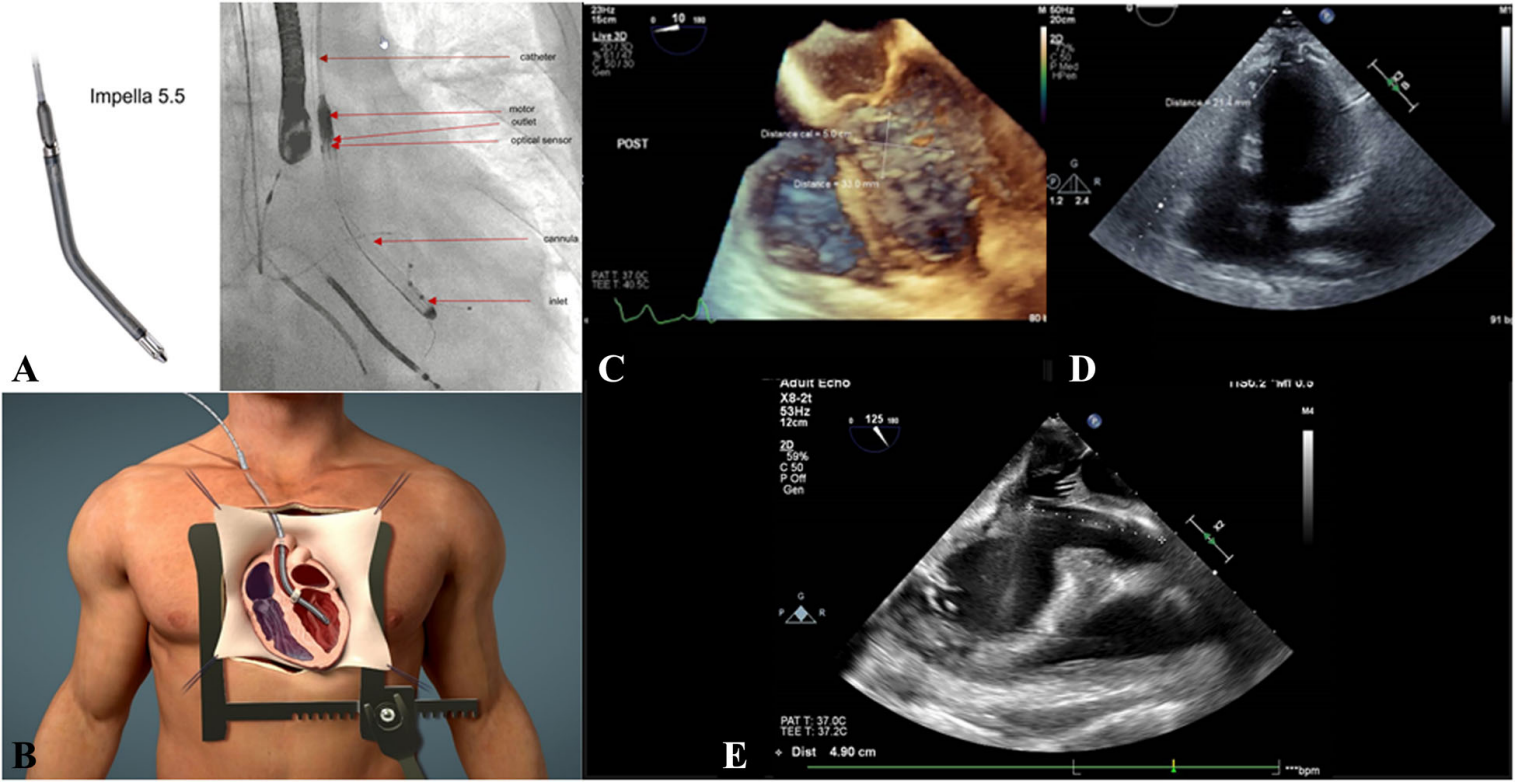
Fig. 1

Ventricular septal defect (VSD) with cardiogenic shock remains a devastating complication following acute myocardial infarction (MI). Despite advances in surgical techniques and aggressive therapy including ventricular assist devices (VAD), the overall morbidity and mortality is frustratingly high. The timing of surgical intervention is critical. Moreover, no guidelines have been established for patient selection for early use of VADs in this setting. The Impella 5.5 with SmartAssist (Abiomed, Danvers, MA) is a surgically implanted temporary VAD recently approved by the FDA for treatment of patients in cardiogenic shock, and may fill a technology gap for perioperative patients who require acute circulatory support **(**Fig. 1a,b). This report summarizes our clinical experience with two patients with post-MI VSD in cardiogenic shock.

## Case presentation

### Patient 1

A 59-year-old man presented to our institution with acute inferior MI and low blood pressure. Examination revealed a loud systolic murmur. Transthoracic echo demonstrated a large posterior VSD with a significant shunt (Qp/Qs ratio of 3.3), right ventricular dysfunction, severe pulmonary arterial hypertension, and akinesia of the inferior wall. Severe mitral valve (MV) regurgitation was observed. Coronary angiography displayed normal left main stem, 100% RCA occlusion, and 40% mid LAD stenosis. RCA has diffuse lesions with severe distal disease and was not graftable. Cardiac index was 1.7 and a chest X-ray revealed pulmonary edema. The patient was diagnosed with inferior MI and a large posterior VSD with cardiogenic shock (< 80/40 mmHg), and underwent urgent VSD repair.

Under cardiopulmonary bypass (CPB) with cardioplegic arrest, left ventriculotomy was made. A posterior VSD measuring 3.5 × 4.5 cm in diameter was found (Fig. 1c). The septal myocardium surrounding the VSD was necrotic. Double patch technique to close VSD was used and several stitches were placed in the MV annulus, correcting the mitral insufficiency. A 10 mm graft was anastomosed to the distal ascending aorta. The Impella was introduced using manual manipulation and transesophageal echocardiography (TEE) guidance, prior to weaning patient from CPB. The device’s 145° angle assisted reposition the device away from the MV apparatus and VSD patch (Supplementary video).


**Video** S1

**Figure 2:**
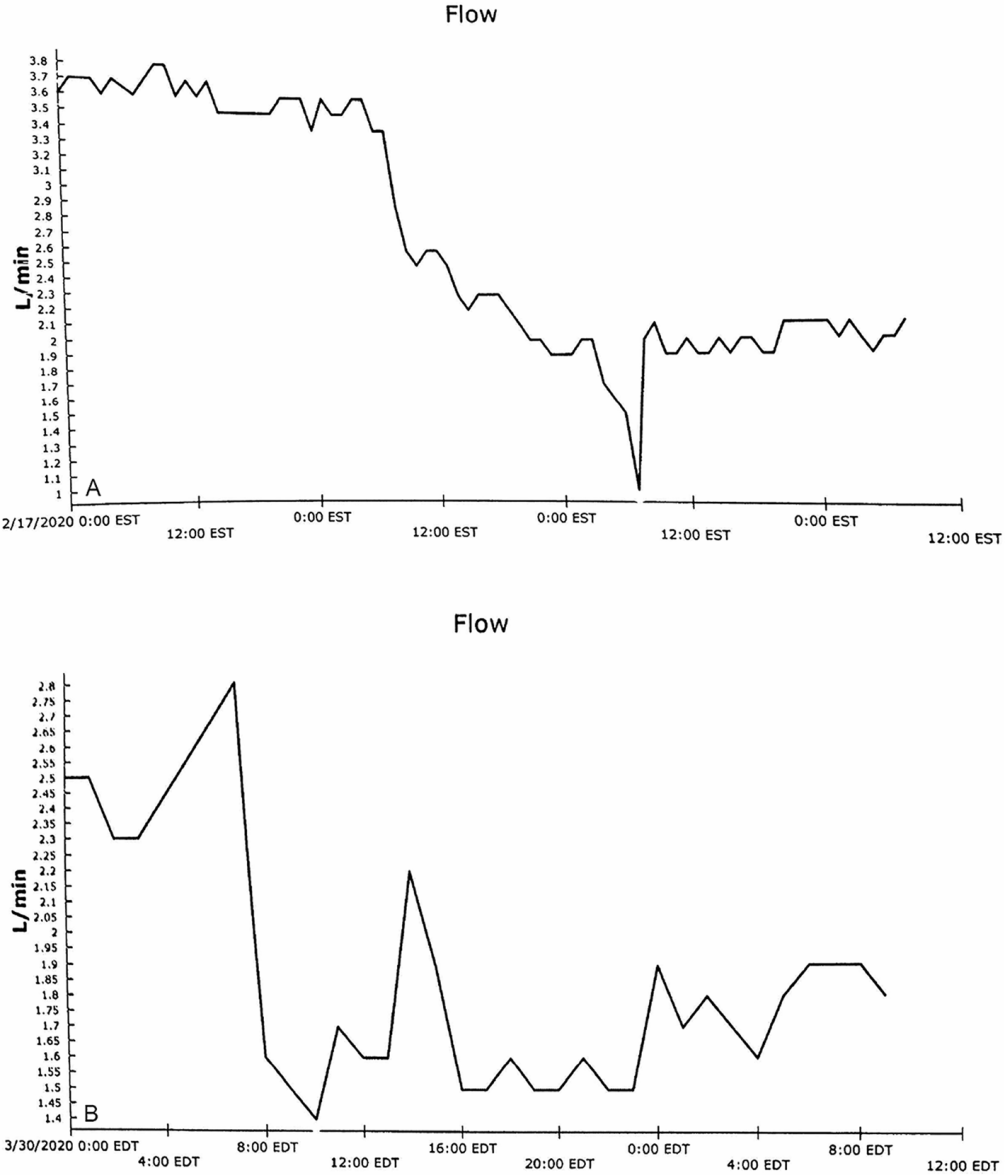
Fig. 2

Minimal postoperative support was required (epinephrine at a dose of 0.02 μg/kg per min). Right ventricular (RV) load declined, as depicted by decreasing RV dimensions (Table 1). The device wean was guided by cardiac power output as Impella flow was reduced (Fig. 2). Impella removal did not require redo sternotomy. The graft was flushed with saline, clamped, trimmed short, over sewn, and left in the subcutaneous space behind the sternal notch. The patient was discharged on postoperative day (POD) 10 and his condition remained stable 14 months later. Follow-up echocardiogram 1 year after septal defect closure showed no residual shunt, good LV function and mild mitral regurgitation.

**Table 1:** Pre- and post-operative hemodynamic parameters

	Preoperative hemodynamic parameters	Pre-discharge hemodynamic parameters
Cardiac index, ml/min/m^2^	1.7	3.2
Qp/Os	3.3	1
EF, %	25	52
LV EDP, mmHg	23	15
RV LTD, mm	88	75
RV MCD, mm	44	35
PAP systolic, mmHg	72	35
PAWP, mmHg	54	18

*EF* ejection fraction; *LV EDP* left ventricular end-diastolic pressure; *Qp/Qs* pulmonary to systemic flow ratio; *RV LTD* right ventricular longitudinal diameter; *RV MCD* right ventricular mid-cavity diameter; *PAP* pulmonary artery pressure; *PAWP* pulmonary artery wedge pressure

### Patient 2

A 60-year-old male was admitted for massive anterior MI with cardiogenic shock (CS). Work-up showed proximal LAD occlusion, a large anterior VSD with a left-right shunt (Qp/Qs ratio of 2.8) and ejection fraction of 25%. Despite intra-aortic balloon counter pulsation and inotropic support, the patient developed shock with cardiac index of 1.8 and serum lactate of 6 mmol/L. In the operating room, a ventriculotomy was performed. The VSD (2.5 × 2.8 cm; Fig. 1d) was repaired with a Bovine pericardial patch and CABGx1 (LIMA to LAD) was carried out. The Impella 5.5 was implanted using the technique described above (Fig. 1e). The SmartAssist technology recognized “ventricularization” of the device (right position) upon arrival in the ICU and repositioning resulted in recovery of hemodynamics. The patient was discharged with a life vest on POD 14, remaining asymptomatic and well on virtual thirteen-month follow-up visits.

## Discussion

Cardiogenic shock on presentation and preoperative deterioration of hemodynamics are strong predictors of mortality [1, 2]. Although AHA guidelines recommend early surgical repair regardless of hemodynamic status, the timing of surgery and use of VADs in the setting of post-MI VSD with cardiogenic shock remains controversial and should be individualized [3]. The main challenge in treating patients with post-MI VSD is whether to correct the defect immediately or to delay surgical closure and allow time for tissue and organ recovery [2]. For our patients in cardiogenic shock, we opted for early surgical repair of VSD with intraoperative Impella placement to allow immediate LV decompression with continuous drainage of LV, thus maintaining flow from LV to the aorta, preventing worsening of cardiac performance and organ malperfusion, while resolving the pulmonary to systemic shunt [4]. We believe that this technique protects the integrity of the VSD patch, providing additional time for septal tissue maturation, and it gives surgeons the opportunity to perform concomitant interventions (i.e, CABG, valve repair/replacement etc.).

In the recent years, the peripheral arterial approach in implanting catheter-based VADs has become the predominant technique [5]. Due to the urgency in cardiogenic shock in our patients, we placed the device through a graft sewn to the distal ascending aorta during CPB after VSD closure; a strategy more time sensitive than an axillary or femoral approach. Moreover, trans-aortic Impella placement in such cases is beneficial for LV venting and continued support allowing for early bed mobilization of the patients compared to femoral access.

Extracorporeal membrane oxygenation (ECMO) is commonly used in such patients; however, ECMO has been shown to compromise LV contractile function with increased LV end-diastolic pressure, LV afterload, impaired myocardial blood flow and the frequent need for an additional intervention such as LV venting. In cases of advanced cardiogenic shock with severe RV dysfunction and multi-organ failure, we have previously utilized ECMO with Impella support. The Impella 5.5 SmartAssist provides more practical and effective postoperative treatment and mechanical support and could facilitate post-operative treatment in patients with LV dysfunction with myocardial stunning. In the case of significant RV failure, one should consider adding RVAD, avoiding the need for ECMO, especially in the lack of severe systemic malperfusion.

With a forward flow to the systemic circulation of up to 6.2 L/min the Impella 5.5 is best able to decompress the heart of all currently available mechanical circulatory support devices used in the setting of cardiogenic shock. This is particularly important in patients with large body habitus or in cardiogenic shock with a vasodilatory component, which may be exacerbated by suboptimal LV decompression. Other features of the Impella 5.5 SmartAssist include the potential for device removal and repositioning at bedside; furthermore, in our cases we observed an improvement in RV function with resolution of pulmonary edema. These findings may lower the decision threshold for an early surgical approach and the initiation of mechanical circulatory support restoring the hemodynamics, preventing the aggravation of organ failure and potentially leading to improved outcomes in patients with post-infarction VSD in cardiogenic shock.

## Conclusions

To our knowledge, this is the first report of surgical implantation of the Impella 5.5 SmartAssist for patients with post-infarct VSD in cardiogenic shock. Our experience suggests the feasibility of using the Impella 5.5 SmartAssist to stabilize the patient, lowering pulmonary arterial and wedge pressure, reduce mitral valve incompetence and support the stunned ventricle with improvement of cardiac index after early surgical VSD closure. This strategy allows for heart recovery while maintaining distal organ perfusion. Our findings concerning the unique utility of the Impella 5.5 in the setting of cardiogenic shock in patients with post-MI VSD merit broader exploration. We should carefully evaluate these critically ill patients. Depending on the degree of right ventricular failure and systemic malperfusion, we should consider other circulatory support strategies.

## Data Availability

Not applicable.

## Abbreviations

VSD: Ventricular septal defect
Post-MI: Post-myocardial infarction
VAD: Ventricular assist device
CPB: Cardiopulmonary bypass
MV: Mitral valve
LV: Left ventricle
POD: Postoperative day
LAD: Left anterior descending artery
ECMO: Extracorporeal membrane oxygenation
